# Holotranscobalamin Is a Useful Marker of Vitamin B12 Deficiency in Alcoholics

**DOI:** 10.1100/2012/128182

**Published:** 2012-03-12

**Authors:** Alberto Fragasso, Clara Mannarella, Angela Ciancio, Oronzo Scarciolla, Nicoletta Nuzzolese, Rocco Clemente, Eustachio Vitullo, Andrea Sacco

**Affiliations:** ^1^Hematology Unit, Ospedale Madonna delle Grazie, ASM, Matera, Italy; ^2^Laboratory Unit, Ospedale Madonna delle Grazie, ASM, Matera, Italy; ^3^Internal Medicine Unit, Ospedale Madonna delle Grazie, ASM, Matera, Italy

## Abstract

*Background*. Measurement of serum cobalamin (Cbl) levels is the standard investigation for assessing vitamin B12 deficiency. Falsely increased values of Cbl can be caused by alcoholic liver disease. Measurement of total vitamin B12 serum levels might be misleading in alcoholics, because a tissue metabolic deficiency is possible even with normal serum Cbl levels. Holotranscobalamin (HoloTC), the Cbl metabolically active fraction, is considered as a better index of vitamin B12 deficiency. *Methods*. For assessing vitamin B12 status, we evaluated 22 adult alcoholic male patients by measuring in parallel serum Cbl, serum folate and red blood cell folate levels, HoloTC levels by the AxSYM assay. *Results*. HoloTC values were reduced in 3 alcoholics with borderline-low Cbl values. Significant positive correlations were found between serum Cbl and HoloTC levels, serum Cbl and gamma-glutamyl transpeptidase (GGT). *Conclusion*. HoloTC measurement is a useful option for assessing vitamin B12 status in alcoholics, particularly in the subjects with borderline Cbl values and may be considered an early marker of vitamin B12 deficiency.

## 1. Introduction

Measurement of total serum cobalamin (Cbl) is the standard screening test for assessing vitamin B12 deficiency, but a diagnostic “gold standard” for this purpose is still lacking, especially in cases with borderline values. A vitamin B12 deficiency increases the concentration of total plasma homocysteine (tHcy) and methylmalonic acid (MMA), while folate deficiency only increases the concentration of tHcy. Many authors recognize tHcy and MMA as the most sensitive and early indicators of vitamin B12 and folate status [1, 2]. In these studies, the two metabolic markers are more specific than serum Cbl levels; however, this opinion is not unanimous [3, 4]. Vitamin B12 in serum is bound to proteins called transcobalamin (TC): most cobalamin is carried on TC I, also called haptocorrin (HC), and 20–30% is carried on TC II. The TC II-cobalamin complex is called holotranscobalamin (HoloTC), that is, the metabolically active fraction. The HoloTC RIA is the first available method for measurement of HoloTC [5]; recently, an automated assay for measuring HoloTC on the Abbott AxSYM analyzer has been introduced [6]. HoloTC, or “active” B12, contains the biologically available Cbl; several studies have shown that HoloTC is the earliest and most specific marker of vitamin B12 deficiency [7, 8]. Falsely increased Cbl values are caused by liver diseases [9]; particularly elevated serum vitamin B12 levels were found in alcoholics with liver disease [10, 11]. In a previous paper, we have shown that some alcohol-dependent patients with megaloblastic anemia may respond to vitamin B12 treatment despite normal cobalamin serum levels [12]. These findings suggest that alcohol consumption may cause falsely normal Cbl serum levels in these patients. In order to evaluate the vitamin B12 status, we have measured the HoloTC levels in 22 alcohol-dependent patients and investigated the association between this parameter, serum Cbl, and other markers of alcohol abuse.

## 2. Patients and Methods

Twenty actively drinking male patients with alcohol dependence (DSM-IV) were evaluated. All patients had a complete blood count, performed by a Sysmex XE-2100 automated analyzer (Sysmex, Kobe, Japan), and routine biochemistry (Synchron LX20 Pro, Beckman Coulter, Brea, Calif, USA). Serum creatinine concentrations were in the normal range in all patients, but one. In parallel were measured serum Cbl levels (normal range: 200–1100 pg/mL), serum folate (normal range: 3.1–24 ng/mL), and RBC folate levels (normal range: 342–786 ng/mL) by the ADVIA Centaur chemiluminescence assays (Siemens Medical Solution Diagnostics, Tarrytown, NY, USA). HoloTC levels were measured by performing the AXSYM assay (Abbot Laboratories, Abbot Park, Ill, USA), which is based on the microparticle enzyme immunoassay (MEIA). We used an automated assay for HoloTC that can be used on the Abbott AXSYM immunoassay analyser. This method is a 2-step sandwich microparticle enzyme immunoassay. In the first step, a HoloTC-specific antibody immobilized in latex microparticles captures HoloTC in samples of serum. In step 2, the captured HoloTC is detected with a conjugate of alkaline phosphatase and anti-TC antibody. In details, the 4-methylumbelliferyl phosphate is added. The conjugate labelled with alkaline phosphatase catalyzes the removal of phosphate from the substrate, generating the fluorescent 4-methylumbelliferyl. This product is finally measured by the optical method MEIA. According to the literature, a reference interval of 40–200 pmol/L was considered as appropriate [13, 14]. Two patients were HCV positive, none was HbSAg positive. HoloTC was also measured in a nonalcoholic 84-years-old patient with pernicious anemia (PA). The diagnosis of vitamin B12 deficiency was based on the following criteria: serum Cbl levels <200 pg/mL, serum folate normal or increased levels, and RBC folate normal or decreased levels. In patients with low or borderline-low serum Cbl (values between 201 and 300 pg/mL, gray zone), reduced HoloTC levels confirmed the diagnosis of vitamin B12 deficiency. In patients with normal or decreased serum Cbl levels, decreased RBC folate levels, and normal or decreased serum folate levels, a diagnosis of isolate folate deficiency was made. In patients with all these parameters decreased, a diagnosis of combined deficiency was made.

### 2.1. Statistical Data Analysis

Results are presented as median and range. Pearson correlation analysis and unpaired *t*-test were used. A *P* value less than 0.05 was considered statistically significant. The statistical procedures were performed with SPSS statistical software v11 (SPSS Inc., Chicago, Ill, USA).

## 3. Results

Age, hematologic, and metabolic characteristics of the patients are shown in Table 1. Serum folate and RBC folate levels was decreased in 10 and 7 patients, respectively; serum Cbl were low in one patient (196 pg/mL), borderline low in 2 patients (201 pg/mL and 275 pg/mL, resp.), and increased (>1100 pg/mL) in 3 patients. Median serum Cbl levels were in the normal range. Reduced HoloTC levels were found in all patients with low-borderline Cbl levels (Table 2). The patient with 275 pg/mL serum Cbl levels also showed a folate deficiency. In the patient with PA, both serum Cbl and HoloTC levels were very low (63 pg/mL and 3.9 pmol/L, resp.). Out of 22, 14 patients were anemic (Hb < 12.5 g/dL). Anemia was severe (Hb < 8 g/dL) in 3 patients, caused by hemorrhagic gastritis in a patient and multifactorial in the other two (folate deficiency and infective disease, thalassemia and chronic renal insufficiency, resp.); in the remaining ones, a mild anemia was linked to the alcohol abuse, associated to a folate deficiency in 4 patients. A significant positive correlation was found between serum Cbl and HoloTC levels (*r* = 0.64, *P* = 0.01), serum Cbl and GGT (*r* = 0.49, *P* = 0.05); serum Cbl and HoloTC levels were inversely, but not significantly, related with MCV. No significant correlations were found between serum Cbl and HoloTC with serum ferritin, AST and ALT levels.

## 4. Discussion

Measurement of serum Cbl concentration, serum folate levels, and RBC folate levels has been the cornerstone for assessing suspected cases of these vitamins' deficiency; however, there are major limitations with this approach. Falsely increased Cbl values are caused by myeloproliferative disorders, liver diseases, intestinal bacterial overgrowth, congenital TC II deficiency, nitrous oxide, and other particular clinical and laboratory circumstances [9, 15]. Increased MMA and tHcy together can be found with primary metabolic defects, renal insufficiency, and hypovolemia, while tHcy alone can increase in alcohol abuse, folate, and vitamin B6 deficiency; MMA is considered a sensitive marker of vitamin B12 deficiency, but the test has a limited availability. Furthermore, in the ambulatory care setting not only Cbl, but also MMA and tHcy levels fluctuate with time and neither predict nor preclude the presence of Cbl-responsive hematologic or neurologic disorders [16]. Several authors have suggested that HoloTC is the better index of Cbl deficiency [7, 17] and the earliest marker for vitamin B12 deficiency in populations at risk [18] and in the elderly [19]. In a previous paper about megaloblastic anemic patients, we found falsely normal serum Cbl levels only in alcoholics [12]; out of 101 adult patients with megaloblastic anemia, normal Cbl serum levels, normal serum, and RBC folate levels were found only in 3 patients, all alcohol dependent, while in another, alcoholic borderline vitamin B12 serum levels were found. All the four patients responded to cobalamin treatment. In this study, we have evaluated the vitamin B12 status in 22 alcoholics by measuring not only Cbl concentrations, serum folate and RBC folate levels, but also HoloTC levels, with the Abbott AxSYM assay. It has been reported that liver diseases may cause an increase in circulating Cbl [10, 11, 20]. In our study, median serum Cbl levels were 480 pg/mL; in many reports, serum Cbl levels were found higher in alcoholics than in the control group but generally remain in the reference range [21, 22]. A significant positive correlation was found between vitamin B12 and GGT, a marker of recent alcohol abuse, as reported by others [20, 23]. With increasing alcohol-related hepatocellular damage, serum Cbl tends to be higher; increased serum vitamin B12 titres correlate with disease severity, and declining levels were found during remission of the disease [24]. This phenomenon is likely caused by Cbl release during hepatic cytolysis and/or a defective storage that causes vitamin B12 to leak out of the liver into circulation; on the other hand, a diminished concentration of TCII and a reduced clearance of HC may be the result of an impaired synthesizing liver capability [10–20]. Therefore, measurement of total vitamin B12 serum levels might be misleading in alcoholics, because a tissue metabolic deficiency is possible even with normal serum Cbl levels. A significant positive correlation was found between serum Cbl and HoloTC levels, as reported by others using the same immunoenzymatic assay [13, 25]. Reduced HoloTC levels were found in 3 alcohol-dependent patients with borderline-low Cbl levels. The patient with clinical signs of vitamin B12 deficiency (pernicious anemia) showed very low levels of HoloTC. Therefore, HoloTC measurement was useful to identify a suspected vitamin B12 deficiency in alcoholic patients, despite borderline Cbl levels. Our data, despite the small sample number, support the assumption that HoloTC is an early marker for diagnosing B12 deficiency. This is a preliminary report: further confirmation needs to be clarified with more cases. As expected, in our series, folate deficiency was the most common problem (out of 22, we found RBC folate low in 7 patients), but also Cbl deficiency is a nonnegligible matter in these patients. Our findings suggest that in alcoholics caution is urged in the interpretation of these vitamin assays, particularly in the subjects with borderline-low Cbl values. In this subset of patients, HoloTC measurement may be a useful option for assessing vitamin B12 status.

## Figures and Tables

**Table 1:** Alcoholic patients: sex, age, hematologic, and metabolic characteristics; *N* = 22 (M 22); mean age (range): 60 years (35–82).

	Normal value	Range	Median
Hb g/dL	12.5–16.5	6–15.3	11.7
MCV fL	80–98	74–110	98.6
Ferritin ng/mL	33–322	48–4841	551
AST U/l	<37	11–251	43
ALT U/l	<40	12–305	34
GGT U/l	5–37	31–2452	120
Cbl pg/mL	200–1100	196–3768	480
Folate serum ng/mL	3.1–24	0.3–8.5	4.1
Folate RBC ng/mL	342–786	147–1193	400
HoloTC pmol/L	>35	21–640	96

Hb: hemoglobin; MCV: mean corpuscular volume; Ferritin: serum ferritin; AST: aspartate aminotransferase; ALT: alanine aminotransferase; GGT: gamma-glutamyl transpeptidase; Cbl: cobalamin; RBC: red blood cells; HoloTC: holotranscobalamin.

**Table 2:** HoloTC in alcoholics with low-borderline Cbl levels.

	Cbl pg/mL	HoloTC pmol/L
Pz 1	196	40
Pz 2	201	21
Pz 3	275	36

HoloTC: holotranscobalamin; Cbl: cobalamin.
